# Continuous-Flow Pulsed Electric Field Treatment of Potato Fiber: Effects on Microbial Load, Physicochemical Properties, and Functional Characteristics

**DOI:** 10.3390/foods15193415

**Published:** 2026-09-24

**Authors:** Rohollah Sadeghi, Obediah C. Rattray, Owen M. McDougal

**Affiliations:** Department of Chemistry and Biochemistry, College of Arts & Sciences, Boise State University, Boise, ID 83725, USA; rohollahsadeghi@boisestate.edu (R.S.); obierattray@u.boisestate.edu (O.C.R.)

**Keywords:** potato fiber, pulsed electric field, microbial reduction, WHC, OHC, fiber microstructure

## Abstract

Potato fiber is a valuable coproduct of potato starch processing, but residual starch and microbial load can limit its quality and application as a food ingredient. This study evaluated continuous-flow pulsed electric field (PEF) treatment for non-thermal microbial reduction and modification of potato fiber properties. Initial batch experiments demonstrated limited microbial inactivation at low electric-field strengths, whereas higher field strengths improved microbial reduction. Continuous-flow treatment at 24 kV/cm was subsequently evaluated at specific energies of 50 and 100 kJ/L, classified as PEF50 and PEF100 respectively. PEF100 resulted in an approximate 1.6-log (96%) reduction in aerobic plate count (APC), greater than 2.5-log (99%) reduction in yeast, and greater than 1 log (97%) reduction in mold. PEF100 also significantly reduced residual starch from 31.7% to 26.8% and increased estimated fiber content from 54.9% to 59.3%. Water-holding capacity was not significantly reduced by PEF treatment, while oil-holding capacity increased modestly following PEF100 treatment. Ash content was unaffected, whereas protein content decreased from 5.8 to 4.7% following PEF treatment. The findings of this study demonstrate that continuous-flow PEF treatment at 100 kJ/L effectively reduced the microbial load and residual starch of potato fiber while maintaining, and in some cases improving, key physicochemical and functional properties.

## 1. Introduction

The potato (*Solanum tuberosum* L.) is one of the most widely cultivated food crops globally and serves as a major raw material for producing French fries, chips, dehydrated products, and starch. These processing operations generate substantial quantities of side streams rich in starch, protein, and dietary fiber that are often underutilized despite their potential value as food ingredients [1,2]. Growing consumer demand for sustainable, clean-label, and fiber-enriched foods has increased interest in recovering and valorizing potato processing coproducts as functional ingredients [3,4]. Potato fiber is particularly attractive because of its high dietary fiber content, excellent water-holding capacity (WHC), oil-holding capacity (OHC), swelling properties, and neutral flavor profile, making it suitable for a wide range of food applications including baked goods, meat products, snacks, and plant-based foods [5,6].

Despite its functional and nutritional benefits, commercialization of potato fiber remains challenging due to microbial contamination associated with wet processing and handling operations. Potato fiber is typically recovered from aqueous processing streams following starch extraction and often contains high moisture levels that promote microbial growth. Elevated microbial loads can negatively affect ingredient safety, shelf life, storage stability, and overall product quality. In commercial manufacturing environments, microbial contamination may also increase drying requirements, energy consumption, and processing costs while creating challenges for ingredient transportation and storage. Therefore, effective microbial reduction strategies are essential for producing safe, high quality, longer-lasting, and commercially viable potato fiber ingredients.

Conventional microbial control methods commonly rely on thermal processing and intensive drying operations. Although effective, these approaches can adversely affect ingredient quality by promoting color development, structural degradation, nutrient losses, and undesirable changes in functional properties. Thermal treatments may alter cell wall architecture, reduce hydration capacity, and negatively impact physicochemical and functional properties of fiber, including water absorption capacity, particle structure, rheological behavior, and polysaccharide composition of dietary fibers in food systems [7,8,9]. Thermal treatment of potato fiber slurries presents significant challenges for achieving microbial reduction and safety while preserving ingredient functionality. Potato fiber suspensions typically exhibit high water-holding capacity, elevated viscosity, and heterogeneous particle structures that can impede heat transfer and reduce temperature uniformity throughout the matrix, creating localized regions where microorganism populations flourish [10,11]. Furthermore, the high moisture-binding capacity of potato pulp and fiber influence heating behavior and processing efficiency, requiring more intensive thermal treatments to achieve target microbial reductions. Thermal processing may also modify pectin and cellulose structures within potato fiber, leading to changes in hydration characteristics and ingredient performance in food applications [8,9]. Consequently, optimizing thermal processes for potato fiber slurries requires balancing effective microbial inactivation with the preservation of desirable functional properties critical for ingredient quality and end-use performance. As a result, there is growing interest in non-thermal technologies that can improve microbial load while preserving or enhancing the physicochemical and functional characteristics of fiber-rich ingredients.

Pulsed electric field (PEF) processing has emerged as a promising non-thermal technology for food preservation and process intensification. PEF involves the application of short-duration, high-voltage electrical pulses that induce transmembrane potential differences across biological membranes, leading to pore formation and electroporation [12,13]. The technology has been extensively investigated for microbial inactivation in liquid foods and for enhancing extraction processes in fruits, vegetables, and plant tissues. Compared with thermal treatments, PEF can achieve microbial reduction while minimizing heat-induced damage to food components and preserving quality attributes [13,14].

Beyond microbial inactivation, PEF treatment may induce structural modifications in plant tissues that influence ingredient functionality. The electrical pulses can disrupt cellular integrity, weaken cell wall networks, and increase tissue permeability, resulting in enhanced mass transfer and changes in microstructure [15]. Previous studies have shown that PEF can increase porosity, alter particle morphology, and modify hydration characteristics of plant materials by promoting cell wall disintegration and increasing accessibility of hydrophilic polysaccharides [16,17]. Such structural changes may significantly affect functional properties including water-holding capacity, oil-holding capacity, swelling behavior, solubility, and rheological performance. For dietary fiber ingredients, these modifications may be particularly important because hydration and network-forming properties largely determine their performance in food applications.

PEF treatment has been extensively investigated as a pretreatment in potato processing to improve cutting performance, enhance tissue permeability, and produce smoother cutting surfaces with reduced feathering during French fry and potato chip production [18]. PEF treatment has also been reported to reduce blanching requirements, decrease oil uptake, and mitigate acrylamide formation during subsequent processing [19,20,21]. Notably, the studies described above generally employed low-to-moderate PEF treatment intensities in terms of electric field strength and specific energy input. These findings demonstrate the potential of PEF to modify potato tissue structure and processing behavior; however, the effects are strongly dependent on the treatment intensity and processing conditions.

The relationship between PEF-induced structural modifications and fiber functionality is of considerable industrial interest. Increased porosity and exposure of cell wall polymers may enhance water absorption and swelling properties, potentially improving texture, moisture retention, and stability in food products. Conversely, excessive structural disruption may reduce particle integrity and negatively affect ingredient performance. Therefore, understanding the balance between microbial reduction and preservation of desirable functional properties is essential for successful implementation of PEF technology in fiber ingredient manufacturing.

Although numerous studies have examined the effects of PEF on microbial inactivation and extraction efficiency in plant-based systems, limited information is available regarding its application to potato fiber ingredients. In particular, the effects of PEF treatment on microbial load, physicochemical characteristics, microstructural properties, and functional performance of potato fiber have not been systematically investigated. Such information is needed to evaluate the feasibility of PEF as a processing technology for producing safe, functional, and commercially viable potato fiber ingredients.

The purpose of this study was to evaluate the impact of pulsed electric field treatment on the microbial load, physicochemical properties, and functional characteristics of potato fiber. Emphasis was placed on understanding how PEF-induced microstructural modifications influence hydration behavior and ingredient functionality while achieving microbial reduction. The findings provide new insights into the application of PEF as a non-thermal processing technology to enhance the safety, quality, and value of potato fiber ingredients and support sustainable utilization of potato processing coproducts.

## 2. Materials and Methods

### 2.1. Potato Fiber Preparation

Fresh potato tubers (cv. Gogu Valley) were obtained from Genesis Organics (Glenn’s Ferry, ID, USA). The potatoes were soaked in tap water for 15 min and washed to remove soil, dirt, and debris. The potatoes were chopped to ~2 cm^3^ using a knife and cutting board. Then 2.2 kg of chopped potatoes were mixed with 1 L of ice/water plus 60 mL of 5% sodium metabisulfite. The mixture was combined using an industrial blender (Waring Commercial, CB15, Torrington, CT, USA) at high speed for 1 min and transferred into a bucket. This blending step was repeated 10 times to process 22 kg of potatoes for each batch. The bucketed slurry was fed into a decanter (Jiangsu Blovebird Centrifuge Co. LTD, HY-80, Zhangjiagang, China) at 40 L/h flowrate, and the resultant cake (insoluble solids) including starch and fiber was separated from the juice at 6000 rpm. The cake was mixed with water in a ratio of 1 to 5 (cake to water), and the slurry was passed through a shaking Sweco sieve (Sweco, LS18S55, Toronto, ON, Canada) with a screen mesh (Powder Technology Inc., Arden Hills, MN, USA, Mesh TBC145, opening of 119 µm). Each batch included 0.5 kg decanter cake plus 2.5 L of tap water on the screen and washed with 20 L of water using a hose nozzle sprayer. Fiber was collected on the screen and starch plus small amounts of fine fiber passed through the sieve. To improve the fiber purity, a second wash was conducted by blending 1.5 L of the first wash fiber plus 1.5 L of clean water for 30 s. The mixture was then filtered on the same screen and washed with 20 L of water. Finally, a third wash was performed by blending 1.5 L of fiber from the second wash and 1.5 L of clean water for 15 s and passed through the same screen and washed with 20 L of water. This fiber slurry was used as-is for the pulsed electric field treatment.

### 2.2. Pulsed Electric Field (PEF) Treatment

#### 2.2.1. Batch PEF Treatment

Preliminary tests were performed using a PEF Pilot Dual pulsed electric field system (Elea Technology GmbH, Quakenbrück, Germany) equipped with either 8 cm or 1 cm batch cells. Additional information on the batch PEF experiments is available in the Supplementary Materials.

#### 2.2.2. Continuous-Flow PEF Treatment

Potato fiber slurry was treated using a continuous-flow pilot scale pulsed electric field system (PEF-Pilot Dual, Elea Technology GmbH, Quakenbrück, Germany) equipped with a 10 mm internal diameter (DN10) continuous cell containing co-linear stainless-steel electrodes with 1 cm gap size. The conductivity of the fiber slurry was adjusted to 0.7 mS/cm using CaCl_2_ solution (20% *w*/*w*). The product flowrate was adjusted to 80 L/h using a pump (INOXPA Kiber KSFT progressive cavity pump, Plano, TX, USA). A high field strength of 24 kV/cm was used to achieve the most effective microbial reduction. A monopolar rectangular shaped pulse of 6 µs duration was used for the PEF treatment. The specific energy was adjusted to 0 (control), 50 kJ/L and 100 kJ/L. The Elea system and software perform calculations to adjust the specific energy that applies to the sample in the continuous-flow cell. The system measures current intensity of each pulse using a sensor, then it adjusts the frequency based on flowrate, voltage, current intensity and pulse width to maintain a consistent specific energy throughout the sample run. Preliminary experiments were conducted using the batch system, and the results indicated that a field strength of 24 kV/cm and a specific energy of 100 kJ/L provided significant microbial reduction in the fiber slurry. At 100 kJ/L, the product outlet temperature reached 50–55 °C. For microbial analysis, samples were collected immediately after passing through the PEF treatment cell, placed on ice, and immediately transferred to Analytical Laboratories Inc. (Boise, ID, USA) for microbial analysis. The potato fiber slurry after the PEF cell was transferred to a 5-gallon bucket containing 10 L tap water to quickly cool down the fiber to room temperature. Control samples were collected by going through the conductivity adjustment with CaCl_2_ salt and passing through the PEF system without any PEF application. All three samples (control, 50 kJ/L, and 100 kJ/L) were pumped through the PEF system at 80 L/hr and then separated by sieve with washing by water to remove added salt used for conductivity adjustment. The washed fiber was placed on freeze dryer trays, frozen for 24 h at −20 °C and loaded into a freeze-dryer (USALab, USA-SFD-8, Xi’an, China) for 48 h. The freeze-dried fiber was blended in a magic bullet blender for 20 s and collected for further analysis. A preliminary trial was performed with the continuous cell, where the conductivity was adjusted to 1.4 mS/cm with KCl solution (see the details and the results of microbial reduction in the Supplementary Materials Figure S3). The results of this preliminary trial demonstrated that microbial suppression could be achieved with either KCl or CaCl_2_ to adjust the conductivity to 1.4 mS/cm or 0.7 mS/cm, respectively.

### 2.3. Microbial Analysis

Microbial analyses were performed by an external accredited laboratory (Analytical Laboratories Inc., Boise, ID, USA) following standard methods and appropriate dilutions. Aerobic plate count and coliform/*E. coli* analyses were performed according to AOAC Official Methods 990.12 and 991.14, respectively [22]. *Salmonella* and *Listeria* were analyzed using AOAC Research Institute Performance Tested Methods 100201 [23] and 020401 [24], respectively. Yeast and mold counts were determined according to Chapter 21 of the Compendium of Methods for the Microbiological Examination of Foods [25].

### 2.4. Water Holding Capacity (WHC)

WHC was performed based on the method of Luo et al., (2017) with minor modifications [26]. Briefly, around 0.5 g of the sample was weighed in a 50 mL centrifuge tube and the mass was recorded, then 30 mL of DI water was added to the fiber and thoroughly mixed for 1 min. The mixture remained on the bench for 24 h to ensure complete hydration, at which time it was centrifuged at 3000× *g* for 20 min at 20 °C (Eppendorf, 5920R, Hamburg, Germany). The supernatant was drained, and the tube was inverted for 2 min to drain any loose water from the tube. The wet or hydrate fiber weight was recorded and WHC was measured according to Equation (1) as grams of water absorbed by one gram of fiber. Each sample was assessed in triplicate.

(1) $$Water\;Holding\;Capacity\;\left(WHC\right)=\frac{Wet\;fiber\;weight\;after\;hydration\;\left(g\right)-Initial\;fiber\;weight\;(g)}{Initial\;fiber\;weight\;(g)}$$

### 2.5. Oil Holding Capacity (OHC)

OHC was performed based on the method of Luo et al., (2017) with minor modifications [26]. Briefly, around 1.0 g of sample was weighed in a 50 mL centrifuge tube and the mass was recorded, then 40 mL of sunflower oil was added to the fiber. The mixture was vortexed for 15 s every 5 min for 20 min followed by centrifugation at 3000× *g* for 25 min at 20 °C (Eppendorf, 5920R, Hamburg, Germany). The excess oil was decanted and drained, then the tube was inverted for 2 min to remove any free oil. The fiber and oil weight were measured and recorded to calculate the oil holding capacity according to Equation (2), as grams of oil entrapped in one gram of fiber. Each sample was assessed in triplicate.

(2) $$Oil\;Holding\;Capacity\;(OHC)=\frac{Fiber\;plus\;entrapped\;oil\;weight\;\left(g\right)-Initial\;fiber\;weight\;(g)}{Initial\;fiber\;weight\;(g)}$$

### 2.6. Total Starch Content

The total starch content of the fiber samples was measured by a Megazyme Total Starch Kit (Megazyme, K-TSTA-100A, Bray, Ireland), which is based on AOAC Method 996.11 and AACC Method 76-13.01.

### 2.7. Moisture Content

The moisture content was measured based on loss-on-drying gravimetric method. Briefly, 1.0 g sample was transferred to an aluminum pan and accurately weighed. The sample was placed in an air-forced oven (Memmert, UF260, Schwabach, Germany) at 105 °C for 16 h to reach a constant weight. After drying, the sample was cooled in a desiccator, the dried materials weight was recorded, and the moisture content was calculated based on mass loss-on-drying.

### 2.8. Ash Content

Briefly, around 0.5 g of each sample was transferred into pre-conditioned crucibles and each weight was recorded. Then the crucible was dried at 105 °C for 6 h to remove moisture. The crucible then was transferred to a muffle furnace (Thermo Fisher Scientific, FB1315M, Asheville, NC, USA) at 550 °C for 6 h until the remaining ash turned white or light grey in color. The crucibles were cooled in a desiccator, and the weight of the ash was recorded.

### 2.9. Crude Protein Content

The crude protein content of the fiber samples was measured by combustion using a LECO nitrogen analyzer (LECO Corporation, FP828P, St. Joseph, MI, USA). Around 100 mg of each sample was placed in the aluminum wraps and loaded to the pre-conditioned and calibrated LECO system. The total nitrogen content was obtained from the system, and it was converted to crude protein content using 6.25 as the conversion factor commonly used for standard regulatory control with crude potato protein content.

### 2.10. Fiber Content

The fiber content was estimated by subtracting total starch content, moisture content, ash content and crude protein content from 100.

### 2.11. Scanning Electron Microscopy

A scanning electron microscope (FEI Teneo field-emission SEM, Hillsboro, OR, USA) was used to monitor the microstructure of the fiber. The freeze-dried samples were sputter-coated with a gold/palladium layer to improve the sample conductivity. The SEM imaging was performed at 5 kV voltage and magnification of 1000× and 8000× to visualize the fiber microstructure.

### 2.12. Statistical Analysis

Statistical analyses were performed using IBM SPSS Statistics version 26 (IBM Corp., Armonk, NY, USA). Data was analyzed using one-way analysis of variance (ANOVA) to evaluate the effect of PEF treatments on the physicochemical, functional, and microbial properties of potato fiber. When significant differences were detected, Tukey’s Honestly Significant Difference (HSD) test was used for pairwise comparisons. Multivariate differences among treatment groups were evaluated using multivariate analysis of variance (MANOVA), with Pillai’s trace used to assess the overall treatment effect. Statistical significance was established at *p* < 0.05.

## 3. Results and Discussion

### 3.1. PEF Effects on Microbial Load of Fiber

Initial experiments were conducted using a batch PEF system to evaluate the effect of electric-field strength and specific energy on the microbial load of potato fiber. Using an 8 cm-gap batch treatment cell (800 mL), the maximum attainable electric-field strength with the ELEA pilot dual system was 3 kV/cm. At this field strength, specific energies of 5, 10, and 20 kJ/L did not result in significant reductions in aerobic plate count (APC) or yeast (Supplementary Materials, Figure S1). A smaller 1 cm-gap batch cell (8 mL) was subsequently used to increase the electric-field strength to 15 kV/cm, which we determined to be our upper limit since higher field strengths resulted in electrical arcing. At 15 kV/cm, APC was reduced by less than 1 log at specific energies of 100 and 150 kJ/L, whereas yeast showed a substantial reduction at both energy levels and was below the detection limit at 150 kJ/L (Supplementary Materials, Figure S2). These results indicated that higher electric-field strength and energy input were required to achieve meaningful microbial reduction.

Based on the batch results, continuous-flow PEF treatment was evaluated using the DN10 treatment cell, which enabled an electric-field strength of up to 24 kV/cm. The continuous-flow system required a minimum flow rate of 80 L/h and a conductivity of approximately 0.2–0.3 mS/cm for stable operation. To enable higher energy input, the conductivity of the potato fiber suspension was adjusted to 0.7 mS/cm using calcium chloride. Potato fiber was then treated at two specific energy levels, 50 and 100 kJ/L, while a control sample was processed through the same system without application of the electric field.

Multivariate analysis demonstrated a significant overall effect of PEF treatment on the microbial profile of potato fiber (Pillai’s trace, *p* < 0.001). Significant treatment effects were also observed for APC (*p* = 0.005), yeast (*p* < 0.001), and mold (*p* < 0.001) (Figure 1). Tukey’s HSD pairwise comparisons showed that APC was significantly lower following treatment at 100 kJ/L compared with the control (*p* = 0.005), whereas the 50 kJ/L treatment did not differ significantly from the control, indicating that higher specific energy was required to achieve substantial APC reduction. Treatment at 100 kJ/L resulted in a 1.6-log reduction in APC, corresponding to approximately 96% reduction, which met the microbial reduction target established for this study. Further microbial reductions may be achieved by increasing the specific energy beyond 100 kJ/L, which can be reached by reducing the fiber slurry flowrate below 80 L/hr and/or increasing the internal diameter of the PEF treatment cell to greater than 10 mm.

Yeast was more sensitive to PEF than APC. Both 50 and 100 kJ/L treatment levels significantly reduced yeast counts, with the 100 kJ/L treatment reducing yeast to below the detection limit (<100 CFU/g), corresponding to a reduction of >2.5 log (>98.8%). The 50 kJ/L treatment also resulted in 1.5-log (96%) reduction in yeast, indicating that this lower-energy treatment may be sufficient when yeast is the primary microbial concern. Mold was similarly reduced by both 50 kJ/L (*p* = 0.026) and 100 kJ/L (*p* < 0.001) compared with the control. Reductions of approximately 0.5-log (70%) and 1.6-log (97%) were observed at 50 and 100 kJ/L, respectively. Overall, yeast showed the greatest sensitivity to PEF, followed by mold and APC.

Comparison of the final potato fiber produced with and without PEF treatment further demonstrated the effectiveness of the process. At 100 kJ/L, PEF resulted in greater than 1.5-log reduction in APC and greater than 2.5-log reduction in yeast compared to the untreated control (Table 1). These results demonstrate the potential of continuous-flow PEF as a non-thermal or minimally thermal approach for reducing microbial loads in potato fiber. Compared with conventional thermal treatment, PEF can achieve microbial inactivation through electroporation during short treatment times, potentially reducing exposure of heat-sensitive food components to elevated temperatures and helping preserve product quality. The continuous-flow configuration also enabled treatment at a substantially higher throughput than the batch-cell experiments, providing a more relevant platform for evaluating PEF under process conditions approaching those encountered during ingredient manufacturing.

Because the potato fiber used in this study did not contain detectable levels of the pathogens *Escherichia coli*, *Salmonella*, or *Listeria monocytogenes*, the efficacy of PEF against these organisms could not be directly evaluated. Future challenge studies using inoculated potato fiber are therefore warranted to determine the effectiveness of PEF against relevant foodborne pathogens and to establish its potential contribution to the microbiological safety of potato-derived ingredients. Liu et al., (2020) used pulsed electric field at different field strengths to reduce *Ent. faecalis* and *E. coli* [27]. They found that PEF and ohmic heating have synergistic effects, which can improve effectiveness of PEF treatment. With ohmic heating effect, PEF treatment at 25 and 30 kV/cm for 1 ms led to 5-log reduction in *Ent. Faecalis*, the specific energy of 831 kJ/L was applied at 25 kV/cm. The specific energy applied in their study was 8.3 times higher than was applied in our research investigation. It is noteworthy to mention that ohmic heating in our PEF100 was significant and increased the fiber slurry temperature to ~50 °C. Temperatures around 50 °C do not provide significant microbial inactivation, but the increase in temperature at PEF100 may affect physicochemical properties, and functional characteristics of potato fiber. Dubey et al. (2023) applied pulsed electric field to rice and black gram fermented batter, where they found around 1.7-log reduction in total bacteria and around 1-log reduction in yeast and mold using 35 kV/cm field strength [28]. They concluded that PEF can effectively inactivate microorganisms without any major changes in the physicochemical properties of batter. In another study, Cserhalmi, et al. (2002) showed that yeasts (*Saccharomyces cerevisiae*) are more sensitive to PEF than *Bacillus cereus* cells [29]. At 20 kV/cm using 10 pulses, *S. cerevisiae* had a 4-log reduction while *B. cereus* cells showed only a 1-log reduction [29]. These findings are consistent with our observations where the order of sensitivity to PEF follows a yeast > mold > APC pattern.

### 3.2. PEF Effects on Physicochemical and Functional Properties of Fibers

Total starch content, WHC, OHC, moisture content, ash content, protein content, and estimated fiber content were evaluated to assess the effects of PEF treatment on the physicochemical and functional properties of potato fiber. Multivariate analysis revealed significant differences among the three treatment groups (control, PEF50, and PEF100) based on their physicochemical and functional properties (Pillai’s trace, *p* < 0.001). The physicochemical and functional properties of potato fiber obtained from the three treatments are presented in Table 2. It is important to note that the observed changes in the physicochemical and functional properties of potato fiber likely resulted from the combined effects of electroporation and ohmic heating. Therefore, the individual contributions of PEF and thermal effects cannot be fully distinguished under the conditions used in this study.

#### 3.2.1. Total Starch

Total starch content differed significantly among the treatments (*p* = 0.04). Tukey’s HSD pairwise comparison showed that the only significant difference was between the control (31.7 ± 2.8%) and PEF100 (26.8 ± 1.1%) treatments (*p* = 0.035). PEF100 reduced the starch content of the potato fiber by approximately 4.9%, corresponding to a relative reduction of approximately 15.5% compared with the control. Considering the fibrous structure of potato fiber and its ability to retain starch granules, this reduction is beneficial for decreasing residual starch in the final fiber ingredient. It is hypothesized that PEF treatment facilitates starch leakage from the fiber structure, which is subsequently removed during further washing and dewatering steps. Although PEF50 also reduced the starch content to 29.7 ± 0.8%, the reduction was not statistically significant compared with the control.

The total starch content observed in the present study was comparable to values reported in the literature. Panasevich et al. (2013) reported a total starch content of 28.7% in raw potato fiber, which is within the range observed in the present study [30]. Ramasamy et al. (2014) investigated the use of enzymatic treatment to facilitate the release of starch granules entrapped within the potato fiber matrix [31]. Treatment with Pectinex Ultra-SP (USP) and a glucanase-rich enzyme preparation (Ebs), particularly their combined application, reduced the amount of starch in potato fiber from approximately 28% to 22% [31]. Although enzymatic treatment can effectively reduce residual starch, its implementation may introduce additional processing and cost considerations, including reaction time, enzyme recovery or inactivation, and potential modification of the fiber matrix. In comparison, PEF provides a relatively nonthermal, physical treatment that may facilitate the disruption of the fiber matrix and improve the release of entrapped starch. The greater starch reduction observed at PEF100 suggests that higher specific energy levels may provide additional starch removal and should be investigated in future studies to optimize the PEF treatment conditions.

#### 3.2.2. Water Holding Capacity (WHC)

Water-holding capacity (WHC) differed significantly among the three treatments (*p* = 0.014). The WHC of potato fiber decreased from 9.9 ± 0.3 g water/g fiber in the control to 8.9 ± 0.3 g water/g fiber following PEF50 treatment, whereas PEF100 resulted in a higher WHC of 11.1 ± 1.0 g/g. However, Tukey’s HSD pairwise comparison showed that neither PEF50 nor PEF100 differed significantly from the control. A significant difference was observed only between PEF50 and PEF100 (*p* = 0.012). Therefore, within the range of PEF conditions evaluated in this study, PEF treatment did not significantly compromise the WHC of potato fiber. The difference between the two PEF energy levels suggests that treatment intensity may influence the hydration properties of the fiber, although additional experiments are needed to confirm this relationship.

The WHC values obtained in this study were comparable to those reported for pressed potato fiber. Ramasamy et al. (2015) reported WHC values ranging from 8.3 to 10.6 mL water/g fiber, which are generally consistent with the values observed in the present study [32]. Differences in reported WHC values among studies can be attributed to several factors, including the characteristics of the raw potato, fiber composition and preparation, drying conditions, particle size, hydration time, and the analytical method used to determine WHC. Notably, Ramasamy et al. (2015) reported that treatment with the glucanase-rich enzyme preparation denoted as Ebs reduced the WHC of potato fiber from 8.3 to 3.3 mL water/g fiber [32]. This substantial reduction suggests that enzymatic modification of the fiber matrix can adversely affect its water-holding properties. In comparison, the present results indicate that PEF treatment, under the conditions evaluated, can reduce residual starch without significantly compromising the WHC of the resulting potato fiber.

#### 3.2.3. Oil Holding Capacity (OHC)

Oil-holding capacity (OHC) differed significantly among the treatments (*p* < 0.001). The OHC decreased from 11.0 ± 0.2 g oil/g fiber in the control to 10.3 ± 0.2 g/g following PEF50 treatment, whereas PEF100 resulted in the highest OHC (11.6 ± 0.1 g oil/g fiber). Tukey’s HSD pairwise comparison showed that the OHC of PEF100-treated fiber was significantly higher than that of the control (*p* = 0.016) although the increase in OHC was relatively small (approximately 5.5% compared with the control). These results indicate that treatment at 100 kJ/L did not compromise the oil-holding functionality of potato fiber and may provide a modest improvement in this functional property. The lack of a significant difference between PEF50 and the control, together with the higher OHC observed following PEF100, suggests that the effect of PEF on OHC may depend on the specific energy applied.

The mechanisms underlying the observed increase in OHC require further investigation. However, several factors may contribute to the higher OHC, including increased specific surface area and porosity, greater exposure of hydrophobic regions, formation of a more loosely structured matrix, changes in particle size and microstructure, and potential alterations in fiber composition and molecular weight [33,34,35]. The OHC values obtained in the present study were substantially higher than those reported in some previous studies. Ma et al. (2022) reported an OHC of approximately 1.5 g oil/g fiber for unmodified potato fiber [36], whereas Jeddou et al. (2016) reported an OHC of 4.4 g oil/g fiber for polysaccharides extracted from potato peel waste [37]. These differences may be associated with differences in fiber composition, degree of purification, particle size, raw material characteristics, processing conditions, and the analytical method used to determine OHC. In particular, the present study evaluated the oil-holding capacity of the whole potato fiber matrix, whereas Jeddou et al. (2016) evaluated isolated polysaccharides, which may have substantially different oil-binding properties [37]. Therefore, direct comparison of absolute OHC values among studies should be made with caution.

#### 3.2.4. Moisture Content and Ash Content

Moisture content increased with increasing PEF treatment intensity. Although all samples were dried under the same conditions using the same freeze-dryer chamber, moisture content increased from 4.0 ± 0.1% in the control to 5.3 ± 0.1% and 5.6 ± 0.0% in the PEF50 and PEF100 treatments, respectively. The higher moisture content following PEF treatment may be associated with changes in the fiber matrix and its water-binding properties resulting from PEF-induced structural modifications. The reduction in residual starch observed following PEF treatment may also contribute to changes in water retention; however, further investigation is required to establish the mechanism responsible for the observed increase in moisture content.

Ash content did not differ significantly among the treatments, indicating that the PEF conditions applied in this study did not substantially alter the mineral content of the final potato fiber. Therefore, the observed effects of PEF on the physicochemical and functional properties of the fiber were not associated with changes in ash content.

The moisture content of potato fiber is commonly reported in the range of approximately 12–14%, depending on the raw material and processing conditions. Commercial potato fiber is typically dried using industrial drying technologies such as spin-flash drying. The substantially lower moisture content observed in the present study is likely attributable to freeze-drying, which provided more extensive moisture removal than would typically be targeted for a commercial fiber ingredient. Therefore, the moisture values obtained in this study should be interpreted in the context of the drying method used.

#### 3.2.5. Protein Content

Protein content differed significantly among the treatments (*p* = 0.002). The control fiber had a protein content of 5.7 ± 0.4%, which was significantly higher than that of both PEF50 (4.4 ± 0.1%, *p* = 0.001) and PEF100 (4.8 ± 0.1%, *p* = 0.011). The reduction in protein content following PEF treatment may be associated with structural changes induced by PEF treatment (combination of electroporation and ohmic effects), which can increase the permeability of cellular structures and facilitate the release or redistribution of proteins from the potato fiber matrix. This release of protein from fiber structure could facilitate protein removal during further washing and dewatering steps and lead to reduction of residual protein in potato fiber. However, changes in the relative composition of the fiber resulting from the reduction in residual starch may also contribute to the observed differences in protein content.

The protein content measured in the present study was comparable to values reported for potato pulp from starch-processing facilities. Bae et al. (2025) reported protein contents ranging from 3.0 to 5.2% in potato pulp [38], which is generally consistent with the values observed in the PEF-treated fiber in the present study.

#### 3.2.6. Estimated Fiber Content

Estimated fiber content was calculated by difference, subtracting the measured moisture, ash, starch, and protein contents from 100%. The estimated fiber content increased from 54.9 ± 2.3% in the control to 56.8 ± 0.8% and 59.3 ± 1.1% following PEF50 and PEF100 treatments, respectively. The difference between the control and PEF100 treatments was statistically significant (*p* = 0.033), whereas the increase observed for PEF50 was not significant. The increase in estimated fiber content following PEF treatment, particularly at 100 kJ/L, is consistent with the reduction in residual starch and protein observed for the PEF100 treatment. Because fiber content was estimated by difference rather than measured directly, the observed increase should be interpreted as a change in the relative composition of the fiber rather than a direct increase in the absolute amount of fiber. These results indicate that PEF100 may improve the compositional purity of the potato fiber by reducing the relative proportion of non-fibrous components, particularly residual starch and protein.

#### 3.2.7. Microstructure of Potato Fiber

Scanning electron microscopy (SEM) was used to characterize the microstructure of the potato fiber and evaluate potential structural changes resulting from PEF treatment. Representative SEM images are presented in Figure 2 and Figure 3. The images show a heterogeneous and irregular fiber matrix composed of cell-wall material with numerous exposed surfaces and structural features that may contribute to interactions with water and other components. Entrapped starch granules were also visible within the fiber matrix.

Visual comparison of the control and PEF100 samples suggested that the PEF100-treated fiber contained smaller structural features and more fragmented cell-wall material in some regions (Figure 2 and Figure 3). However, examination of multiple locations within the fiber samples did not reveal a consistent morphological difference that could be conclusively attributed to PEF treatment. This variability is likely associated with the heterogeneous nature of potato fiber, in which the size, shape, and distribution of cell-wall fragments and entrapped starch granules vary considerably within and among samples. Therefore, although localized structural differences were observed, the SEM analysis did not provide sufficient evidence to establish a uniform morphological modification of potato fiber resulting from PEF treatment. These SEM images provide qualitative visualization of the heterogenous potato fiber structure and presence of entrapped starch granules, not a quantitative measurement to show the difference between control and PEF100 treatment.

## 4. Conclusions and Remarks

This study demonstrated the potential of continuous-flow pulsed electric field (PEF) treatment as a process-relevant, nonthermal/minimally thermal approach for improving the quality of potato fiber. Compared with batch treatment, continuous-flow PEF enabled higher electric-field strength and throughput, resulting in more effective microbial reduction. At 100 kJ/L, PEF reduced aerobic plate count by approximately 1.6 log, yeast by more than 2.5 log, and mold by greater than 1 log, while also reducing residual starch by approximately 15.5% and increasing the relative fiber content of the ingredient. Importantly, these compositional changes were achieved without compromising water-holding capacity, oil-holding capacity, or ash content, with a modest improvement in OHC observed at 100 kJ/L. SEM images suggest localized structural modification, although the heterogeneous nature of potato fiber prevented definitive conclusions regarding PEF-induced morphological changes. Overall, the ability of continuous-flow PEF to simultaneously reduce microbial load and residual starch, while maintaining key functional properties, highlights the potential for PEF to be used in the production of higher-quality potato fiber ingredients. Further research studies focused on optimizing electric-field strength, specific energy, conductivity, residence time, and temperature may reduce energy requirements to achieve microbial reduction and compositional targets. It is also recommended to perform challenge studies by inoculation of potato fiber with pathogens to better demonstrate PEF treatment contribution in products food safety. This work has demonstrated the technical feasibility of the proposed process; however, it has not yet evaluated the challenges associated with industrial-scale implementation, including capital investment requirements, energy consumption, conductivity adjustment costs, and other operational considerations.

## Figures and Tables

**Figure 1:**
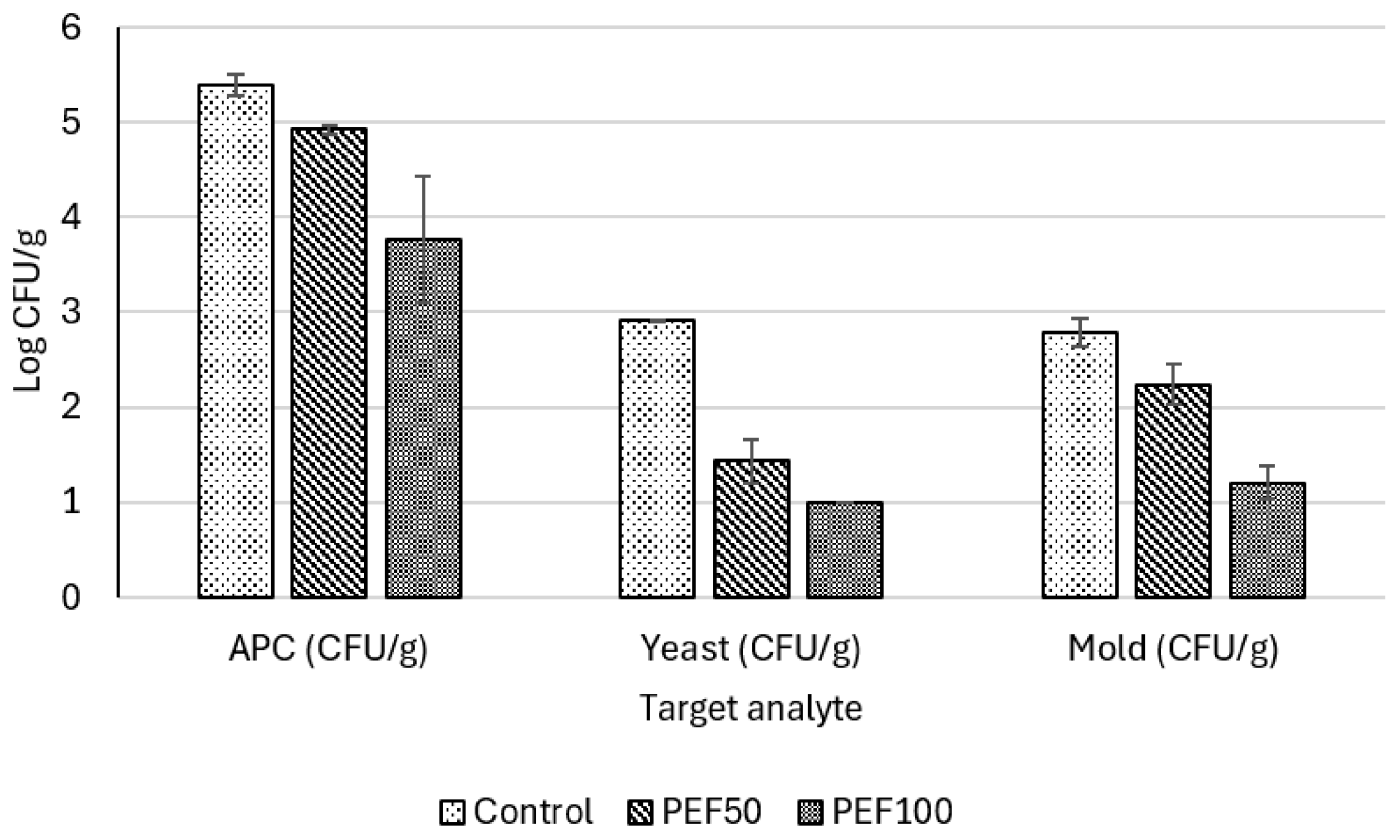
Aerobic plate count (APC), yeast and mold count for potato fiber treated with different levels of pulsed electric field, where no-treatment (Control), 50 kJ/L at 24 kV/cm (PEF50), and 100 kJ/L at 24 kV/cm (PEF100).

**Figure 2:**
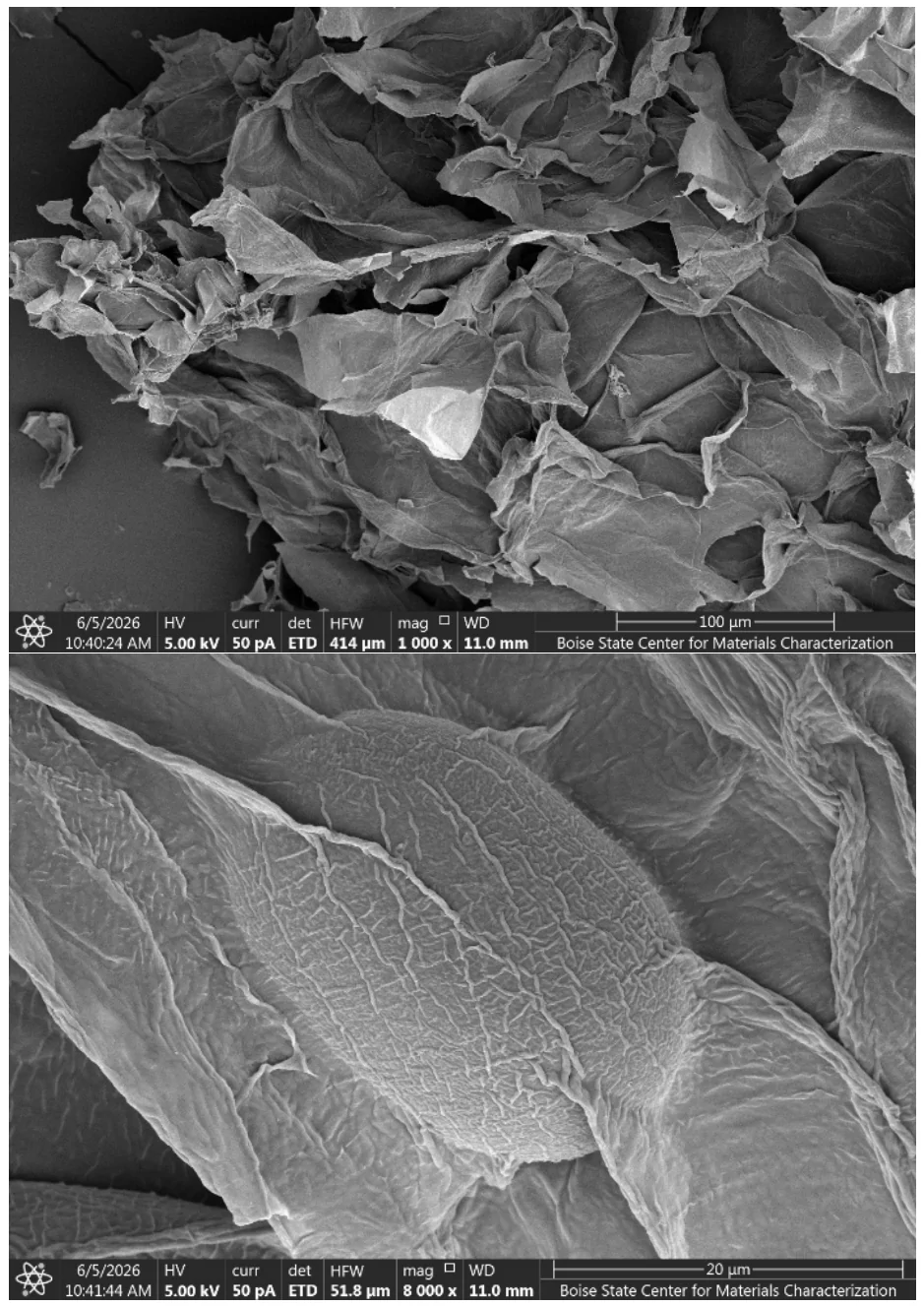
SEM images of potato fiber without pulsed electric field treatment (control), the right-hand side image shows a starch granule trapped inside the cell wall matrix.

**Figure 3:**
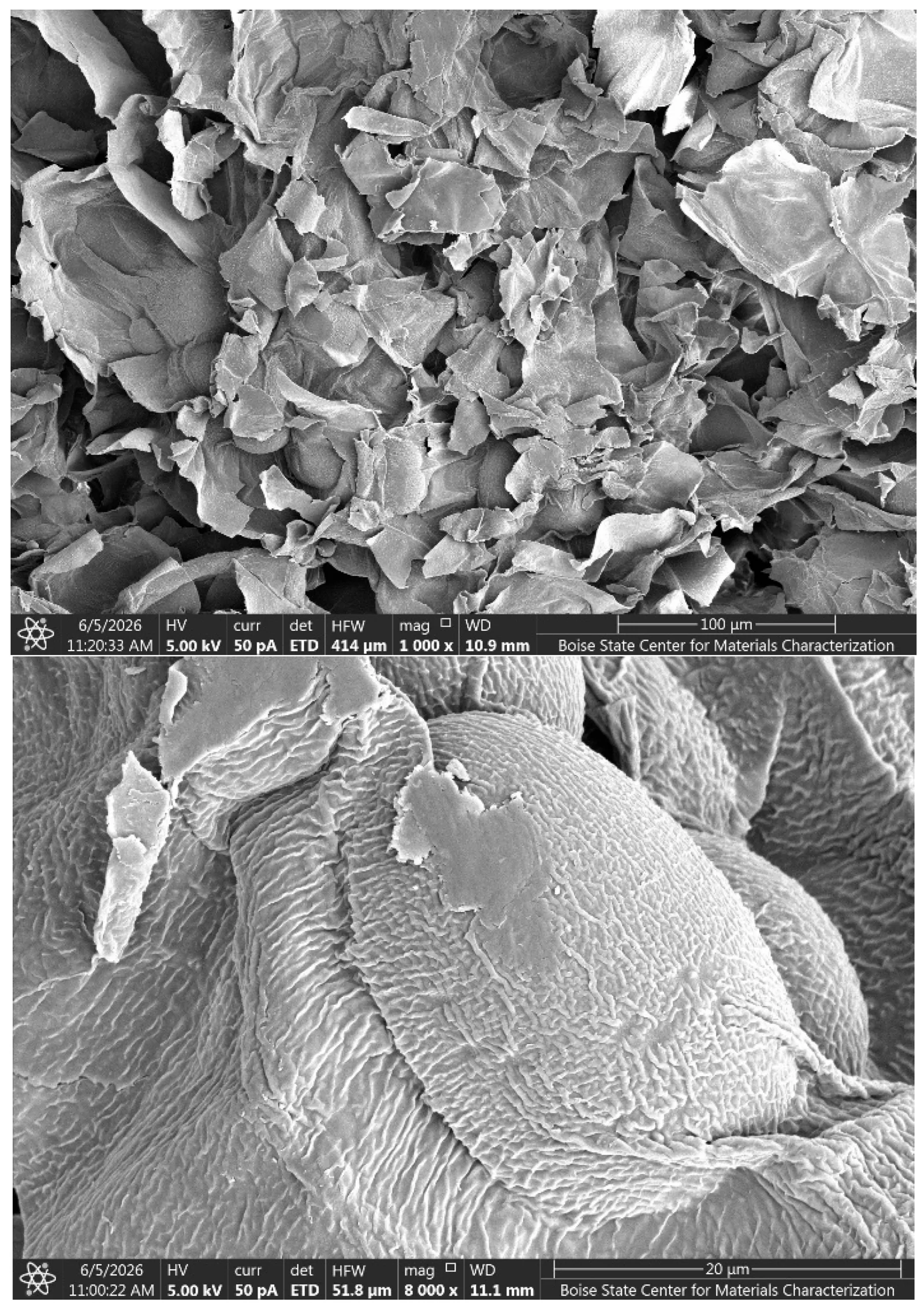
SEM images of potato fiber processed with pulsed electric field treatment at 100 kJ/L specific energy (PEF100); the image on the right shows a starch granule trapped inside the cell wall matrix.

**Table 1:** Comparison between the microbial load of dried potato fiber produced without pulsed electric field treatment (Control) and with 100 kJ/L specific energy at 24 kV/cm field strength treatment (PEF100).

Microorganisms	Control (CFU/g)	PEF100 (CFU/g)
Aerobic plate count (APC)	380,000	11,000
*Escherichia coli*	<100	<100
Yeast	31,000	<100
Mold	<100	<100
*Salmonella*	Undetected in 25 g	Undetected in 25 g
*Listeria monocytogenes*	Undetected in 25 g	Undetected in 25 g

**Table 2:** Physicochemical and functional properties of potato fiber made by no PEF treatment (control) and PEF treated at 50 kJ/L (PEF50) and 100 kJ/L (PEF100).

Treatment	WHC (g Water/g Fiber)	OHC (g Oil/g Fiber)	Starch (%)	Moisture (%)	Ash (%)	Protein (%)	Estimated Fiber (%)
Control	9.9 ± 0.3 ^ab^	11.0 ± 0.2 ^b^	31.7 ± 2.8 ^a^	4.0 ± 0.1 ^c^	3.6 ± 0.1 ^a^	5.7 ± 0.4 ^a^	54.9 ± 2.3 ^b^
PEF50	8.9 ± 0.3 ^b^	10.3 ± 0.2 ^c^	29.7 ± 0.8 ^ab^	5.3 ± 0.1 ^b^	3.8 ± 0.0 ^a^	4.4 ± 0.1 ^b^	56.8 ± 0.8 ^ab^
PEF100	11.1 ± 1.0 ^a^	11.6 ± 0.1 ^a^	26.8 ± 1.1 ^b^	5.6 ± 0.0 ^a^	3.6 ± 0.2 ^a^	4.8 ± 0.1 ^b^	59.3 ± 1.1 ^a^

Different lowercase letters within the same column indicate significant differences among treatments (*p* < 0.05).

## Data Availability

The original contributions presented in this study are included in the article and its Supplementary Materials. Further inquiries regarding the data can be directed to the corresponding author.

## Supplementary Materials

The following supporting information can be downloaded at: https://www.mdpi.com/article/10.3390/foods15193415/s1, Figure S1: Aerobic plate count (APC) and yeast count of potato fiber with different levels of pulsed electric field including no-treatment (control), 5 kJ/kg, 10 kJ/kg and 20 kJ/kg specific energy at 3 kV/cm field strength, Figure S2: Aerobic plate count (APC) and yeast count of potato fiber with different levels of pulsed electric field including no-treatment (control), 50 kJ/kg, 100 kJ/kg and 150 kJ/kg specific energy at 15 kV/cm field strength. <LOD: below limit of detection, Figure S3: Aerobic plate count (APC), yeast and mold count of potato fiber with different levels of pulsed electric field including no-treatment (control), 50 kJ/kg, and 100 kJ/kg specific energy at 24 kV/cm field strength in continuous flow. <LOD: below limit of detection.

## References

[1] Gustavsson J., Cederberg C., Sonesson U., Van Otterdijk R., Meybeck A. (2011). Global Food Losses and Food Waste: Extent, Causes and Prevention. https://www.fao.org/4/mb060e/mb060e00.pdf.

[2] Schieber A., Saldaña M.D. (2009). Potato Peels: A Source of Nutritionally and Pharmacologically Interesting Compounds—A Review. http://www.globalsciencebooks.info/Online/GSBOnline/images/0906/FOOD_3(SI2)/FOOD_3(SI2)23-29o.pdf.

[3] Sharma S.K., Bansal S., Mangal M., Dixit A.K., Gupta R.K., Mangal A. (2016). Utilization of food processing by-products as dietary, functional, and novel fiber: A review. Crit. Rev. Food Sci. Nutr..

[4] Vescovo D., Manetti C., Ruggieri R., Spizzirri U.G., Aiello F., Martuscelli M., Restuccia D. (2025). The valorization of potato peels as a functional ingredient in the food industry: A comprehensive review. Foods.

[5] Warren F.J., Fukuma N.M., Mikkelsen D., Flanagan B.M., Williams B.A., Lisle A.T., Cuív P.Ó., Morrison M., Gidley M.J. (2018). Food starch structure impacts gut microbiome composition. Msphere.

[6] Mudgil D., Barak S. (2013). Composition, properties and health benefits of indigestible carbohydrate polymers as dietary fiber: A review. Int. J. Biol. Macromol..

[7] Elleuch M., Bedigian D., Roiseux O., Besbes S., Blecker C., Attia H. (2011). Dietary fibre and fibre-rich by-products of food processing: Characterisation, technological functionality and commercial applications: A review. Food Chem..

[8] Bengtsson H., Wikberg J., Tornberg E. (2011). Physicochemical characterization of fruit and vegetable fiber suspensions. II: Effect of variations in heat treatment. J. Texture Stud..

[9] Trabert A., Schmid V., Keller J., Emin M.A., Bunzel M. (2022). Chemical composition and technofunctional properties of carrot (*Daucus carota* L.) pomace and potato (*Solanum tuberosum* L.) pulp as affected by thermomechanical treatment. Eur. Food Res. Technol..

[10] Bellara S.R., Fryer P.J., McFarlane C.M., Thomas C.R., Hocking P.M., Mackey B.M. (1999). Visualization and modelling of the thermal inactivation of bacteria in a model food. Appl. Environ. Microbiol..

[11] Hassani M., Condon S., Pagán R. (2007). Predicting microbial heat inactivation under nonisothermal treatments. J. Food Prot..

[12] Toepfl S., Heinz V., Knorr D. (2006). Applications of pulsed electric fields technology for the food industry. Pulsed Electric Fields Technology for the Food Industry: Fundamentals and Applications.

[13] Barba F.J., Parniakov O., Pereira S.A., Wiktor A., Grimi N., Boussetta N., Saraiva J.A., Raso J., Martin-Belloso O., Witrowa-Rajchert D. (2015). Current applications and new opportunities for the use of pulsed electric fields in food science and industry. Food Res. Int..

[14] Buckow R., Ng S., Toepfl S. (2013). Pulsed Electric Field Processing of Orange Juice: A Review on Microbial, Enzymatic, Nutritional, and Sensory Quality and Stability. Compr. Rev. Food Sci. Food Saf..

[15] Puértolas E., Luengo E., Álvarez I., Raso J. (2012). Improving Mass Transfer to Soften Tissues by Pulsed Electric Fields: Fundamentals and Applications. Annu. Rev. Food Sci. Technol..

[16] Lebovka N.I., Praporscic I., Vorobiev E. (2004). Effect of moderate thermal and pulsed electric field treatments on textural properties of carrots, potatoes and apples. Innov. Food Sci. Emerg. Technol..

[17] Luengo E., Álvarez I., Raso J. (2013). Improving the pressing extraction of polyphenols of orange peel by pulsed electric fields. Innov. Food Sci. Emerg. Technol..

[18] Fauster T., Schlossnikl D., Rath F., Ostermeier R., Teufel F., Toepfl S., Jaeger H. (2018). Impact of pulsed electric field (PEF) pretreatment on process performance of industrial French fries production. J. Food Eng..

[19] Zhang C., Ye J., Lyu X., Zhao W., Mao J., Yang R. (2022). Effects of pulse electric field pretreatment on the frying quality and pore characteristics of potato chips. Food Chem..

[20] Ostermeier R., Hill K., Dingis A., Töpfl S., Jäger H. (2021). Influence of pulsed electric field (PEF) and ultrasound treatment on the frying behavior and quality of potato chips. Innov. Food Sci. Emerg. Technol..

[21] Santiago-Mora P., Skinner M., Hendricks A., Rimkus T., Meyer B., Gratzek J., Pu S., Woodbury L., Bond L., McDougal O. (2024). Pulsed electric field effect on acrylamide reduction and quality attributes of continuous-style Lamoka potato chips. Heliyon.

[22] Chemists A.A., Horwitz W. (1990). Official Methods of Analysis, Vol. I.

[23] Peng L.X., Wallace M., Andaloro B., Fallon D., Fleck L., Delduco D., Tice G. (2011). Modification of the BAX System PCR assay for detecting Salmonella in beef, produce, and soy protein isolate. Performance Tested Method 100201. J. AOAC Int..

[24] Juck G., Gonzalez V., Allen A.-C.O., Sutzko M., Seward K., Muldoon M.T. (2018). Romer Labs RapidChek^®^
*Listeria monocytogenes* Test System for the Detection of *L. monocytogenes* on Selected Foods and Environmental Surfaces. J. AOAC Int..

[25] Ryu D., Wolf-Hall C. (2013). 21. Yeasts and Molds. Compendium of Methods for the Microbiological Examination of Foods.

[26] Luo X., Wang Q., Zheng B., Lin L., Chen B., Zheng Y., Xiao J. (2017). Hydration properties and binding capacities of dietary fibers from bamboo shoot shell and its hypolipidemic effects in mice. Food Chem. Toxicol..

[27] Liu X., Lendormi T., Le Fellic M., Lemée Y., Lanoisellé J.-L. (2020). Hygienization of mixed animal by-product using pulsed electric field in a continuous treatment system: Synergistic effect with ohmic heating on the inactivation of indicator bacteria. Waste Manag..

[28] Dubey P.K., Kumar K.S., Rawson A., Anandakumar S., Baskaran N., Thamburaj S. (2023). Effect of pulsed electric field on physicochemical properties of rice and black gram fermented batter. J. Food Process Eng..

[29] Cserhalmi Z., Vidacs I., Beczner J., Czukor B. (2002). Inactivation of *Saccharomyces cerevisiae* and *Bacillus cereus* by pulsed electric fields technology. Innov. Food Sci. Emerg. Technol..

[30] Panasevich M., Rossoni Serao M., de Godoy M.R.C., Swanson K.S., Guérin-Deremaux L., Lynch G., Wils D., Fahey G., Dilger R.N. (2013). Potato fiber as a dietary fiber source in dog foods. J. Anim. Sci..

[31] Ramasamy U.R., Lips S., Bakker R., Gruppen H., Kabel M.A. (2014). Improved starch recovery from potatoes by enzymes and reduced water holding of the residual fibres. Carbohydr. Polym..

[32] Ramasamy U.R., Gruppen H., Kabel M.A. (2015). Water-holding capacity of soluble and insoluble polysaccharides in pressed potato fibre. Ind. Crops Prod..

[33] Sztupecki W., Rhazi L., Depeint F., Aussenac T. (2023). Functional and Nutritional Characteristics of Natural or Modified Wheat Bran Non-Starch Polysaccharides: A Literature Review. Foods.

[34] Aslan Türker D., Işçimen E.M. (2025). Insoluble dietary fiber and microparticle formation from olive pomace: Effects on emulsification and interfacial behavior in Pickering emulsions. J. Food Meas. Charact..

[35] Wang S., Fang Y., Xu Y., Zhu B., Piao J., Zhu L., Yao L., Liu K., Wang S., Zhang Q. (2022). The effects of different extraction methods on physicochemical, functional and physiological properties of soluble and insoluble dietary fiber from Rubus chingiiHu. fruits. J. Funct. Foods.

[36] Ma Q., Ma Z., Wang W., Mu J., Liu Y., Wang J., Stipkovits L., Hui X., Wu G., Sun J. (2022). The effects of enzymatic modification on the functional ingredient-Dietary fiber extracted from potato residue. LWT.

[37] Jeddou K.B., Chaari F., Maktouf S., Nouri-Ellouz O., Helbert C.B., Ghorbel R.E. (2016). Structural, functional, and antioxidant properties of water-soluble polysaccharides from potatoes peels. Food Chem..

[38] Bae J.-E., Baik M.-Y., Lee S., Cao L., Kim H.-S. (2025). Physicochemical properties of hydrolysate and residues from HCl-hydrolyzed potato pulp. Food Sci. Biotechnol..

