# Supplementary material for: Continuous-Flow Pulsed Electric Field Treatment of Potato Fiber: Effects on Microbial Load, Physicochemical Properties, and Functional Characteristics
Source: Foods. 2026 Sep 24;15(19):3415. doi: 10.3390/foods15193415 (PMC13664005; doi:10.3390/foods15193415)
Supplement: Supplementary file 1 [file foods-15-03415-s001.zip › foods-4558911-supplementary.pdf]

Supplementary Materials

Batch evaluation of microbial inactivation in potato fiber

Batch 1: The first batch experiment was conducted to evaluate the effectiveness of pulsed electric field (PEF) treatment for reducing the microbial load of potato fiber. An 8-cm-gap batch treatment cell with an 800 mL working volume was coupled to the Elea pilot dual system. Because the electrode gap was 8 cm and the maximum available voltage was 24 kV, the maximum achievable electric-field strength was 3 kV/cm. Three specific energy levels (5, 10, and 20 kJ/kg) were evaluated at 3 kV/cm using potato fiber without any modification of its conductivity (<0.2 mS/cm). The batch system was not subject to the conductivity limitations encountered with the continuous-flow system, thus, no salt was added to the samples. Figure S1 shows that none of the PEF treatments resulted in a measurable reduction in aerobic plate count (APC) or yeast compared with the untreated control.

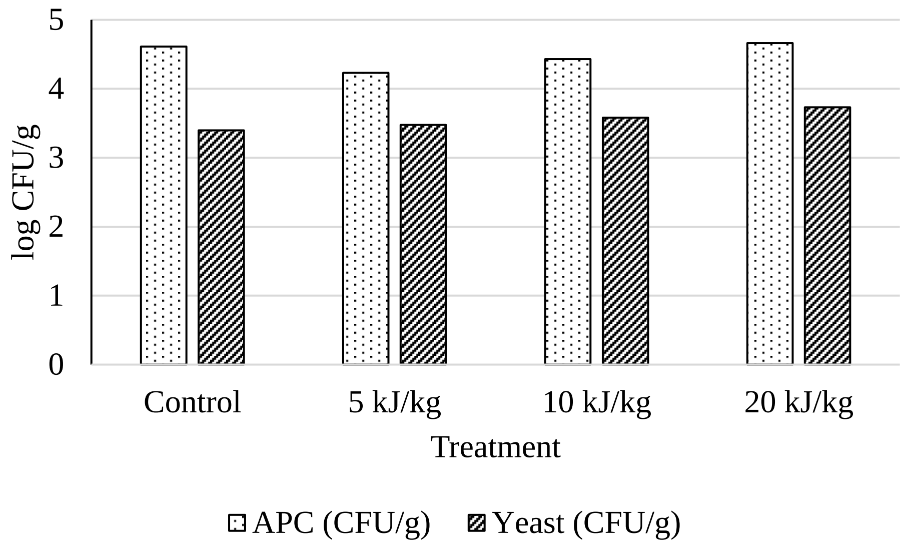

Figure S1. Aerobic plate count (APC) and yeast count of potato fiber with different levels of pulsed electric field including no-treatment (control), 5 kJ/kg, 10 kJ/kg and 20 kJ/kg specific energy at 3 kV/cm field strength.

Batch 2: To evaluate the effect of higher electric-field strength on microbial reduction, a smaller batch treatment cell with a 1-cm electrode gap and approximately 15 mL working volume was used. This configuration theoretically allowed the electric-field strength to reach 24 kV/cm; however, arcing occurred at higher voltages, and a maximum field strength of 15 kV/cm was therefore used without significant arcing. At 15 kV/cm, the specific energy was increased to 50, 100, and 150 kJ/kg by adjusting the

treatment conditions, including the number of pulses. The combination of the higher electric-field strength (15 kV/cm) and higher specific energy levels (100 and 150 kJ/kg) resulted in measurable reductions in both APC and yeast, with yeast showing greater sensitivity to the treatment. At 150 kJ/kg, the yeast count decreased below the detection limit of 100 CFU/g. These results, together with the limited microbial reduction observed in Batch 1, indicated that higher electric-field strength and specific energy were required to achieve meaningful microbial inactivation. Based on these findings, subsequent experiments were conducted using the continuous-flow treatment system, which allowed operation at higher field strengths and specific energies. It is important to note that the initial microbial load in Batch 2 was lower than that observed in the other experimental batches, which may have influenced the magnitude of the observed microbial reductions.

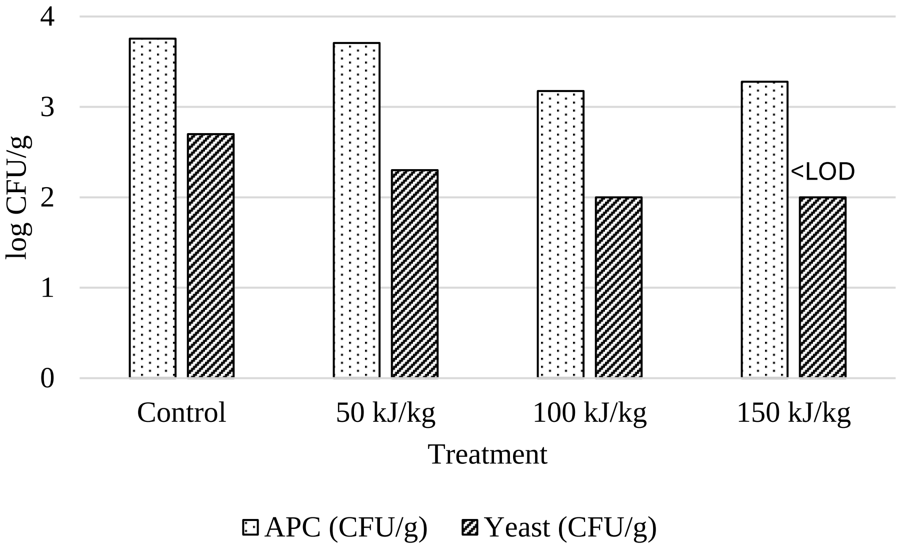

Figure S2. Aerobic plate count (APC) and yeast count of potato fiber with different levels of pulsed electric field including no-treatment (control), 50 kJ/kg, 100 kJ/kg and 150 kJ/kg specific energy at 15 kV/cm field strength. <LOD: below limit of detection

Continuous-flow treatment using the DN10 cell: For the continuous-flow trial, the flow rate was set to the minimum measurable flow rate of the system (80 L/h). The initial conductivity of the potato fiber slurry was low (<0.2 mS/cm) and was adjusted to 1.4 mS/cm by adding a 20% KCl solution. The electric-field strength was set to 24 kV/cm, which was the maximum field strength achievable under these operating conditions. At this flow rate, conductivity, and field strength, the maximum specific

energy that could be applied was 100 kJ/L. The effects of PEF treatment on aerobic plate count (APC), yeast, and mold were subsequently evaluated at three treatment levels: untreated control, 50 kJ/L, and 100 kJ/L. Significant reductions in microbial load were observed following PEF treatment, with the 100 kJ/L treatment providing greater reductions than 50 kJ/L. At 100 kJ/L, approximately 4-log, >2-log, and >1-log reductions were observed for APC, yeast, and mold, respectively. These results demonstrated the effectiveness of continuous-flow PEF for microbial reduction and provided the basis for the experimental conditions used in the final continuous-flow trial presented in the main manuscript. Although the overall reduction trends were consistent between this preliminary trial and the experiments presented in the main manuscript, the magnitude of microbial reduction differed. These differences may be associated with variations in initial microbial load, raw potato characteristics, fiber processing conditions, slurry conductivity, and the salt used for conductivity adjustment. Therefore, this trial was considered preliminary and was used to guide the selection and optimization of conditions for the final experiments. The limit of detection (LOD) varied among samples depending on the sample matrix, dilution scheme, and the lowest dilution that could be reliably plated and quantified by the laboratory. Consequently, the reported LOD may differ among experimental runs.

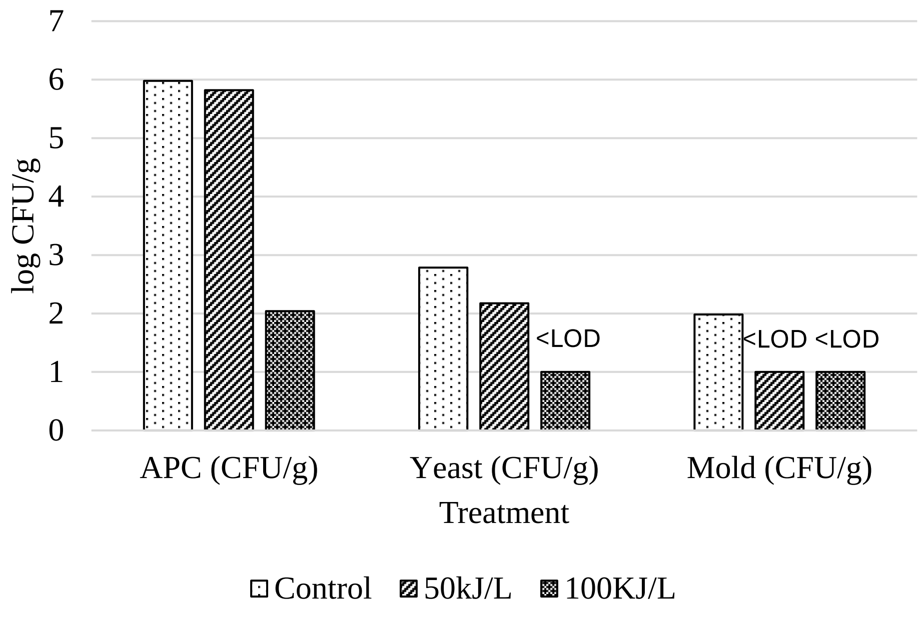

Figure S3. Aerobic plate count (APC), yeast and mold count of potato fiber with different levels of pulsed electric field including no-treatment (control), 50 kJ/kg, and

100 kJ/kg specific energy at 24 kV/cm field strength in continuous flow. <LOD: below limit of detection
